# Comparison of PCR and microscopy for the detection of asymptomatic malaria in a *Plasmodium falciparum/vivax *endemic area in Thailand

**DOI:** 10.1186/1475-2875-5-121

**Published:** 2006-12-14

**Authors:** Russell E Coleman, Jetsumon Sattabongkot, Sommai Promstaporm, Nongnuj Maneechai, Bousaraporn Tippayachai, Ampornpan Kengluecha, Nattawan Rachapaew, Gabriela Zollner, Robert Scott Miller, Jefferson A Vaughan, Krongtong Thimasarn, Benjawan Khuntirat

**Affiliations:** 1 Departments of Entomology and Immunology, U.S. Army Medical Component, Armed Forces Research Institute of Medical Sciences, Bangkok, Thailand; 2 University of North Dakota, Grand Forks, North Dakota, USA; 3 Ministry of Public Health, Nonthaburi, Thailand

## Abstract

**Objective:**

The main objective of this study was to compare the performance of nested PCR with expert microscopy as a means of detecting *Plasmodium *parasites during active malaria surveillance in western Thailand.

**Methods:**

The study was performed from May 2000 to April 2002 in the village of Kong Mong Tha, located in western Thailand. *Plasmodium vivax *(PV) and *Plasmodium falciparum *(PF) are the predominant parasite species in this village, followed by *Plasmodium malariae *(PM) and *Plasmodium ovale *(PO). Each month, fingerprick blood samples were taken from each participating individual and used to prepare thick and thin blood films and for PCR analysis.

**Results:**

PCR was sensitive (96%) and specific (98%) for malaria at parasite densities ≥ 500/μl; however, only 18% (47/269) of *P. falciparum*- and 5% (20/390) of *P. vivax*-positive films had parasite densities this high. Performance of PCR decreased markedly at parasite densities <500/μl, with sensitivity of only 20% for *P. falciparum *and 24% for *P. vivax *at densities <100 parasites/μl.

**Conclusion:**

Although PCR performance appeared poor when compared to microscopy, data indicated that the discrepancy between the two methods resulted from poor performance of microscopy at low parasite densities rather than poor performance of PCR. These data are not unusual when the diagnostic method being evaluated is more sensitive than the reference method. PCR appears to be a useful method for detecting *Plasmodium *parasites during active malaria surveillance in Thailand.

## Background

The detection of asexual parasites by light microscopy of Giemsa-stained thick and thin films remains the standard laboratory method for the diagnosis of malaria [1,2]. Although detection of parasites in symptomatic patients reporting to local malaria clinics is the primary means used for malaria diagnosis in Thailand, use of active (cross-sectional) surveillance provides a tool for detection of patients with asymptomatic malaria and relatively low parasite rates. In Thailand, active surveillance is used in remote areas where individuals may have difficulty in reaching a malaria clinic – in this situation malaria clinic personnel make periodic visits to a given village and examine blood smears from all individuals present in the village. In Thailand and many other malaria endemic regions, there are problems and limitations associated with reliance on microscopic diagnosis of malaria for both active and passive case detection [3-5]. These include lack of skilled microscopists, variation in individual training and/or experience, limited supply of microscopes and reagents as well as variation in equipment maintenance, and inadequate quality control [6]. When symptomatic patients with a relatively high parasitaemia (e.g., >500 asexual parasites/μl) report to a malaria clinic for treatment, microscopy can provide an accurate diagnosis that is used to initiate appropriate chemotherapy. However, accuracy of microscopy can decrease significantly at lower parasitaemia levels [4]. Parasitaemia rates in asymptomatic patients are often quite low – in a recent study in western Thailand, the median asexual parasitaemia rate in 82 *Plasmodium falciparum *and 98 *P. vivax*-positive individuals was 848 and 155 asexual parasites/μl blood, respectively [7].

Polymerase chain reaction (PCR)-based methods have been used since the early 1990's for the detection of *Plasmodium *parasites in human patients. Several experimental assays using various primers and extraction and detection techniques have been reported [8-12]. In general, PCR was more sensitive and specific than examination of thick or thin blood smears, particularly in cases with low parasite rates or mixed infections [2,8,10,13-16]. PCR was also more sensitive than the QBC method [2] and various dipstick assays [17].

Although the majority of studies have shown that PCR is both sensitive and specific for the diagnosis of malaria, there are limitations that can affect the accuracy of the method. Selection of appropriate primers, methods used for collection and storage of blood samples, and extraction methods can all affect PCR performance. Jelinek et al. [18] reported that the sensitivity of PCR was as much linked to parasite density as was microscopy. They found that sensitivity of PCR was affected by both parasite density and by geographic differences in parasite populations. Other studies have reported that PCR may occasionally yield false negative results [9,19,20]. Barker et al. [20] carefully analysed discrepancies between microscopy and PCR, and although true false negative PCR results did occur, the majority of discrepancies resulted from problems with microscopy. Most recently, Scopel et al. [21] determined that use of DNA extracted from thick blood smears resulted in poor detection of malaria parasites, particularly with parasite densities less than 20/μl.

In this study the performance of nested PCR for the detection and identification of *Plasmodium *parasites was evaluated in a malaria endemic village in western Thailand, with expert laboratory microscopy used as the reference standard. The goal of the study was to evaluate performance of PCR at low parasite densities in a population that was primarily asymptomatic.

## Methods

### Study site

The study was performed from May 2000 to April 2002 in the village of Kong Mong Tha (98°33'16" E and 15°10'17" N), located in Laivo Sub-District, Sangkhlaburi District, Kanchanaburi Province, western Thailand. Kong Mong Tha is an isolated village accessible only by boat or foot for 10 months of the year. *Plasmodium vivax *(PV) and *Plasmodium falciparum *(PF) are the predominant parasite species in this village, followed by *Plasmodium malariae *(PM) and *Plasmodium ovale *(PO). During a recent two-year study, 92% (320/346) of parasitemic individuals in this village were asymptomatic at the time blood was collected [22]. The study was approved by the Ethics Committee of the Ministry of Public Health (MOPH), Bangkok, Thailand, and by the Human Subjects Research Review Board of the United States Army, Fort Detrick, Maryland, U.S.A.

### Patients and sample collection

Adults (≥18 years old) and children (≥1 to <18 years old) living in Kong Mong Tha were enrolled in the study. Informed consent was obtained from all adults willing to participate in the study, with a parent/guardian giving consent for children. Three teams of investigators went from house to house during a 4-day period each month that the study was conducted. Each individual was questioned about signs and symptoms of malaria, travel history, and medications taken during the prior two weeks. Fingerprick blood samples were taken from each participating individual and used to prepare thick and thin blood films. An additional 3–5 drops of blood were spotted onto filter paper for subsequent PCR analysis. Isocode^® ^Stix (Schleicher and Schuell BioScience, Inc., Keene, New Hampshire) were used for the collection of blood during the first 5 months of the study and 903 Whatman Filter Paper (Schleicher and Schuell BioScience, Inc., Keene, New Hampshire) thereafter. Both Isocode Stix and Whatman Filter Paper provide an acceptable matrix for the collection of whole-blood samples for subsequent processing for malaria diagnosis by PCR [19,23]. After air-drying for several hours, blood samples were stored at room temperature in double zip-lock plastic bags for subsequent DNA extraction.

### Microscopy

Thick/thin films were stained with 10% Giemsa solution and examined at ×1,000 under oil immersion by an expert microscopist (N.M. or N.R.) with over 10 years of experience. Each microscopist correctly identified the species of *Plasmodium *as well as parasite densities in 100% (50 of 50) of slides in a blinded panel of samples provided prior to the start of the study. The microscopist was blinded to any clinical diagnosis and PCR results during the course of this study. The parasite density was counted per 500 leukocytes and was then expressed as the number of trophozoites per microliter by assuming a leukocyte count of 7,000/μl. Kain et al. [9] found that this value provided a good estimate of leukocyte density for villagers in Western Thailand. The initial thick film was considered negative if no parasites were seen after 500 leukocytes were counted. A high quality (Olympus) microscope with an incandescent light source was used. Each film required approximately 20 minutes to read.

### PCR amplification

DNA was extracted from the filter paper/Isocode Stix samples using the methods described by Plowe et al. [24]. Nested PCR amplification was performed as described by Kimura et al. [25]. Both genus (P1F and P2R) and species specific primers (FR, MR, OR, and VR) were designed from the small subunit ribosomal RNA gene as previously described [25]. Briefly, 200 ng of DNA (0.05 ug for 8,000 wbc in 1 μl of blood) was used as a template in the first amplification step of the nested PCR and 1 μl of a 1:20 dilution of the first PCR product was used for the second amplification. All reactions were performed in a 20 μl volume consisting of 1× PCR Gold buffer II (50 mM of KCl, 15 mM of Tris-HCl, pH 8.0), 1.5 mM of MgCl2, 200 μM of each dNTP, 0.4 μM of each primer (P1F and P2R for the first PCR and P1F and one of each reverse primer–FR, OR, MR, and VR–for the second PCR), and 0.25 unit of AmpliTag Gold (Applied Biosystems, Foster City, CA, USA). The PCR product was resolved by electrophoresis in 2% agarose gels, stained with ethidium bromide, and observed under ultraviolet transillumination. The expected PCR products are 140–160 bp for the first step and 110 bp for the second one. Technicians conducting the PCR were blinded to any clinical diagnosis or microscopy results.

### Treatment of infected patients

The name, age, and house number of all individuals with a malaria-positive blood film were provided to employees of the local Thai Ministry of Public Health (MOPH) malaria clinic. All malaria-positive individuals were treated according to the malaria treatment protocols of the Thai MOPH. Treatment follow-up was provided by Thai MOPH malaria clinic employees and in accordance with Thai MOPH standard procedures.

### Non-concordant results

All samples with non-concordant results were re-evaluated by both microscopy and PCR. The individual reading the blood films or running the PCR was blinded to the initial test results.

### Data analysis

Epi-Info version 6 [26] was used to calculate test performance and acceptability evaluation indices of PCR, with microscopy used as the reference standard. For this analysis, it was assumed that microscopy is always correct. Performance indices followed those used by Tjitra et al. [27] and were calculated for the following microscopic diagnoses: total malaria (all species of *Plasmodium*), PF malaria (to include mixed infections), and PV malaria. Variables measured included the number of true positives (TP), number of true negatives (TN), number of false positives (FP), and number of false negatives (FN). Sensitivity was calculated as TP/(TP + FN), specificity was calculated as TN/(TN + FP), the positive predictive value (PPV) was calculated as TP/(TP + FP), and the negative predictive value (NPV) was calculated as TN/(FN + TN). Test accuracy, the proportion of all tests that gave a correct result, was defined as (TP + TN)/number of all tests. Reliability was expressed as the J index ((TP × TN) - (FP × FN))/((TP + FN)(TN + FP)). Results were considered false positive if microscopy detected PF and the PCR assay detected PV, and visa versa.

In order to evaluate repeatability of each diagnostic method, the performance of each assay were evaluated using data from samples with non-concordant results. Repeatability of each assay was calculated by comparing the second set of test results with the initial test results. Sensitivity and specificity were evaluated using the initial test results as the reference standard.

## Results

A total of 672 individuals (305 females and 367 males) participated in the study. The age range was 1 to 92 years old (mean = 22.1 years; median = 15.3 years). Sixty percent (407/672) of individuals enrolled in the study had a least one microscopy-positive blood film during the 2 years that the study was conducted. Seventy-one percent (291/407) had 1 or 2 positive films, 21% (86/407) had 3 or 4 positive films, and 7% (30/407) had 5 or more positive films.

Of the 8,590 blood films collected over the course of the study, 7.7% (665) were found to have malaria parasites by microscopy. Of the *Plasmodium*-positive slides, 39.8% (265) were PF, 58.0% (386) were PV, 1.5% (10) were PM, and 0.6% (4) were mixed PF/PV infections (Table 1). Of the PV-positive blood films, 79.8% (308/386) had asexual parasites alone, 19.4% (75/386) had both asexual parasites and gametocytes, and 0.8% (3/386) had gametocytes alone. For PF-positive films, 86.0% (228/265) had asexual parasites alone, 13.2% (35/265) had asexual parasites and gametocytes, and 0.8% (2/265) had gametocytes alone, while 91% of PM-positive films had both asexual parasites and gametocytes. The mean density of PF parasites was 892.7/μl (SEM = 164.3), with a range from 28–14,000/μl. For PV, the mean density was 265.5/μl (SEM = 66.5), with a range from 14–14,000/μl, while for PM, the mean density was 324.8/μl (SEM = 128.4), with a range from 28-1,134/μl. Prevalence rates for both PF and PV were highest at the start of the study and in general decreased over the 24 months that the study was conducted.

**Table 1:** Comparison of PCR and expert laboratory microscopy for active malaria surveillance.

	**No. of samples with the following result by PCR (% of total in row)**						
	
**Expert microscopy resμlt**	***P. falciparum***	***P. vivax***	***P. malariae***	***P. ovale***	**Mixed**	**Negative**	**Total**
*P. falciparum*	102 (38.4%)	13 (4.9%)	0 (0.0%)	0 (0.0%)	7 (2.7%)	143 (54.0%) ^a^	265
*P. vivax*	4 (1.0%)	131 (33.9%)	1 (0.3%)	0 (0.0%)	8 (2.1%)	242 (62.7%) ^b^	386
*P. malariae*	0 (0.0%)	0 (0.0%)	8 (80.0%)	0 (0.0%)	0 (0.0%)	2 (20.0%)	10
Mixed	3 (75.0%)	0 (0.0%)	0 (0.0%)	0 (0.0%)	1 (25.0%)	0 (0.0%)	4
Negative	70 (0.9%)	214 (2.7%)	7 (0.1%)	2 (<0.1%)	18 (0.2%)	7,614 (96.1%)	7,925
Total	179 (2.1%)	358 (4.2%)	16 (0.2%)	2 (<0.1%)	34 (0.40%) ^c^	8,001 (93.1%)	8,590

^a ^98% (140/143) of microscopy-positive *P. falciparum *samples that were PCR negative had <250 parasites/μl.

^b ^99% (239/242) of microscopy-positive *P. vivax *samples that were PCR negative had <250 parasites/μl.

^c ^Includes the following mixed infections: 28 *P. falciparum*/*P. vivax*, 3 *P. vivax*/*P. ovale*, 2 *P. vivax*/*P. malariae *and 1 *P. falciparum*/*P. malariae*.

An overall comparison of PCR and expert laboratory microscopy for active malaria surveillance is presented in Table 1. There was 87% (242/278) agreement on the species of parasite present in samples that were positive by both PCR and microscopy; however, a high proportion (58.2%, 387/665) of all microscope-positive samples were negative by PCR and a high proportion (52.8%, 311/589) of PCR-positive samples were negative by microscopy. Ninety-eight percent (381/387) of the microscope-positive, PCR-negative samples had fewer than 250 parasites/μl blood.

Table 2 presents a comparison of PCR with expert microscopy for the detection of PF and PV at various parasite densities. At parasite densities of 500/μl or greater, PCR correctly identified 96% (45/47) of microscopy-positive PF samples and 100% (20/20) of microscopy-positive PV samples; however, only 10% (67/659) of positive films had parasite densities this high. Performance of PCR dropped off markedly with decreasing parasite (both PF and PV) densities, with sensitivity dropping to approximately 20–25% at densities of <100/μl (Table 2). Forty-four percent (118/269) of all PF-positive blood films and 75% (294/390) of all PV-positive films had parasite densities of <100/μl.

**Table 2:** Performance of PCR relative to expert laboratory microscopy at different *Plasmodium falciparum *and *Plasmodium vivax *parasite densities

	***P. falciparum***		***P. vivax***	
		
**Parasites/μl**	**No. detected by microscopy^a^**	**No. positive by PCR (%)**	**No. detected by microscopy^a^**	**No. positive by PCR (%)**
>10,000	10	10 (100.0%)	3	3 (100.0%)
5,000–9,999	4	4 (100.0%)	1	1 (100.0%)
1,000–4,999	22	21 (95.5%)	11	11 (100.0%)
500–999	11	10 (90.9%) ^b^	5	5 (100.0%)
250–499	17	14 (82.4%)	19	15 (78.9%)
100–249	87	29 (33.3%)	57	34 (59.6%)
1–<99	118	24 (20.3%)	294	71 (24.1%)
Negative	8,321	96 (1.2%)	8,200	251 (3.1%)
Total	8,590	208 (2.4%)	8,590	391 (4.6%)

^a ^Data for each parasite includes 4 mixed infections

^b ^A single sample was negative for *P. falciparum *by PCR, however, this sample was positive for *P. vivax *and *P. malariae *by PCR

There was no significant difference (*X*^2 ^test, P < 0.05) between Iso-code Stix and the Whatman Filter Paper for subsequent detection of PF or PV at parasite densities of >100/μl; however, use of the Iso-Code Stix resulted in the detection of significantly (X^2 ^test, P < 0.05) more PV-positive films than did use Whatman filter paper at parasite densities of 1–99/μl and significantly more PF and PV in microscopy negative samples.

When using expert microscopy as the reference standard, PCR was both sensitive and specific for the detection of both PF and PV at parasite densities above 500/μl. However, at parasite densities below 500/μl, sensitivity of PCR dropped off markedly (Table 3). The non-concordance between microscopy and PCR at parasite densities below 100/μl was remarkable (Table 4). A total of 118 samples with fewer than 100 PF parasites/μl blood were detected microscopically. PCR failed to detect 94 (80%) of these samples. Conversely, a total of 120 PF positive samples were detected by PCR – microscopy failed to detect 96 (80%) of these samples. Similar results were obtained with PV samples, with both microscopy and PCR failing to detect 75–80% of positive samples detected by the corresponding method. Although quantitative PCR was not used in this study, it can be assumed that the majority of PCR-positive samples that were microscopy-negative contained fewer than 100 parasites/μl blood.

**Table 3:** Performance characteristics of PCR at different parasite densities relative to expert laboratory microscopy for active surveillance for *Plasmodium falciparum *and *Plasmodium vivax*.^a^

**Trophozoites/μl (total positive)**	**Sensitivity (95% CI)**	**Specificity (95% CI)**	**Positive Predictive Value (95% CI)**	**Negative Predictive Value (95% CI)**	**Accuracy**	**J Index**
			***P. falciparum***			
>5000/μl (14)	100.0% (73.2–100.0)	97.7% (97.4–98.0)	6.7% (3.9–11.3)	100.0% (99.9–100.0)	98%	0.98
>1000/μl (36)	97.2% (83.8–99.9)	98.0% (97.7–98.3)	16.8% (12.1–22.8)	100.0% (99.9–100.0)	98%	0.95
>500/μl (47)	95.7% (84.3–99.3)	98.1% (97.8–98.4)	21.6% (16.4–28.0)	100.0% (99.9–100.0)	98%	0.94
>100/μl (151)	58.3% (50.0–66.2)	98.6% (98.3–98.8)	42.3% (35.6–49.3)	99.2% (99.0–99.4)	98%	0.57
>1/μl (267)	41.6% (35.7–47.8)	98.8% (98.6–99.0)	53.6% (46.6–60.5)	98.1% (97.8–98.4)	97%	0.40
			***P. vivax***			
>5000/μl (4)	100.0% (39.6–100.0)	95.5% (95.0–95.9)	1.0% (0.3–2.8)	100.0% (99.9–100.0)	95%	0.95
>1000/μl (15)	100.0% (74.7–100.0)	95.6% (95.2–96.0)	3.8% (2.2–6.1)	100.0% (99.9–100.0)	96%	0.96
>500/μl (20)	100.0% (80.0–100.0)	95.7% (95.2–96.1)	5.1% (3.2–7.9)	100.0% (99.9–100.0)	96%	0.96
>100/μl (96)	71.9% (61.6–80.3)	96.2% (95.8–96.6)	17.6% (14.1–21.9)	99.7% (99.5–99.8)	96%	0.68
>1/μl (387)	35.9% (31.2–40.9)	96.9% (96.5–97.3)	35.8% (31.1–40.8)	97.0% (96.5–97.3)	94%	0.33

^a ^For this analysis we assume that microscopy is the gold standard (i.e., always correct).

**Table 4:** Relationship between PCR and expert laboratory microscopy with *Plasmodium falciparum *and *Plasmodium vivax *parasite densities of less than 100 parasites/μl.

	***P. falciparum***		***P. vivax***	
		
**Expert Microscopy Resμlt**	**PCR Positive^a^**	**PCR Negative**	**PCR Positive^a^**	**PCR Negative**
Microscopy Positive	24	94	71	223
Microscopy Negative	96	8,225	251	7,949

^a ^Samples with parasite densities greater than 100/μl by microscopy are not included in this analysis.

A total of 698 samples that had non-concordant PCR and microscopy results when initially tested were retested by both microscopy and PCR and the performance of each assay calculated by assuming that the initial test result with each method was correct. The agreement between first and second test results was significantly better for PCR (75.1% sensitivity, 91.9% specificity) than for microscopy (33.5% sensitivity, 58.5% specificity). This data clearly indicates that PCR was the more repeatable method.

## Discussion

Data from this study highlights the problem of using a less-than-perfect diagnostic test as a reference standard. Microscopic results were initially considered as the reference standards for true positive and true negative results, with all subsequent statistical analysis based on this assumption. Although there was good agreement between PCR and microscopy at parasite densities of >500 parasites/μl, the majority of positive blood films in the village of Kong Mong Tha had fewer than 250 parasites/μl blood. There was extremely poor concordance between microscopy and PCR at these relatively low parasite densities. The poor performance of PCR at low parasite densities presumably reflected limitations of microscopy as much as PCR. Many of the PCR results that were considered false-positives and false-negatives for analysis were presumably true-positives and true-negatives (i.e., the microscopy result was incorrect). When PCR was considered the reference standard, the performance of microscopy was just as poor as that observed for PCR when microscopy was used as the reference standard.

Because it was difficult to determine whether microscopy or PCR was the more accurate assay, all non-concordant samples were retested in order to determine which method was the more repeatable test (assuming that the test that was more repeatable was more accurate). A panel of 698 samples that had non-concordant results was retested and the performance of each assay calculated by comparing the results from the second test with those of the initial test (i.e., the initial test was used as the reference standard for statistical purposes). The data from this test clearly demonstrated that PCR was a more repeatable, less subjective test than was microscopy with parasite densities of less than 250/μl. All performance criteria (sensitivity, specificity, PPV, NPV, accuracy and reliability) were much lower for microscopy than for PCR.

The limitations and shortcomings of microscopy are well-documented [1,2], with significant problems existing even in fairly sophisticated laboratories. A rigorous quality assurance program is essential if performance of microscopy is to be maintained at a high level [2]. The two microscopists who examined all blood films in this study have a total of over 35 years of experience reading malaria slides, and had recently completed and passed a quality assurance test developed by the Walter Reed Army Institute of Research (R.S. Miller, personal communication). In spite of having an expert team of microscopists, this study highlights the difficulty in conducting an active surveillance program in areas where infection rates and parasite densities are low. Of the 8,590 blood films that were collected, 98.7% were either negative (7,925) or had fewer than 250 parasites/μl blood (556). Both microscopists examined slides for an average of 12 hours per day for 3 days in a row during each 5-day trip to Kong Mong Tha – it is not surprising that mistakes were made under these conditions (long hours examining mostly negative slides).

## Conclusion

PCR is a less subjective test than is microscopy – this was clearly demonstrated when the set of 698 non-concordant slides was retested. Each performance indicator (sensitivity, specificity, PPV, NPV, accuracy and J index) was markedly higher for PCR than for microscopy. Although sensitivity of PCR can be related to parasite density [17,28], data indicates that PCR is a viable method for conducting active malaria surveillance in western Thailand.

## Authors' contributions

REC, JS and BK were involved in all stages of this study. SP, BT and AK participated in the coordination of the laboratory work. NM, NR and GZ participated in the coordination of the field work. RSM, JAV and KT were involved in the design of the study.

## Disclaimer

The opinions of assertions contained in this manuscript are the private ones of the authors and are not to be construed as the official or reflecting views of the Department of Defense or the Armed Forces Research Institute of Medical Sciences.
